# EGFR: A Master Piece in G1/S Phase Transition of Liver Regeneration

**DOI:** 10.1155/2012/476910

**Published:** 2012-09-23

**Authors:** Alexandra Collin de l'Hortet, Hélène Gilgenkrantz, Jacques-Emmanuel Guidotti

**Affiliations:** ^1^Institut Cochin, Department of Endocrinology Metabolism and Cancer, Université Paris-Descartes, CNRS, UMR8104, 75014 Paris, France; ^2^INSERM, U1016, 75014 Paris, France

## Abstract

Unraveling the molecular clues of liver proliferation has become conceivable thanks to the model of two-third hepatectomy. The synchronicity and the well-scheduled aspect of this process allow scientists to slowly decipher this mystery. During this phenomenon, quiescent hepatocytes of the remnant lobes are able to reenter into the cell cycle initiating the G1-S progression synchronously before completing the cell cycle. The major role played by this step of the cell cycle has been emphasized by loss-of-function studies showing a delay or a lack of coordination in the hepatocytes G1-S progression. Two growth factor receptors, c-Met and EGFR, tightly drive this transition. Due to the level of complexity surrounding EGFR signaling, involving numerous ligands, highly controlled regulations and multiple downstream pathways, we chose to focus on the EGFR pathway for this paper. We will first describe the EGFR pathway in its integrity and then address its essential role in the G1/S phase transition for hepatocyte proliferation. Recently, other levels of control have been discovered to monitor this pathway, which will lead us to discuss regulations of the EGFR pathway and highlight the potential effect of misregulations in pathologies.

## 1. Introduction

Although mammals have almost completely lost the fascinating regeneration capacities of amphibians, their liver retained this unique ability. This process is evolutionarily conserved, presumably because it is critical to mammals' survival. Two-thirds partial hepatectomy (PH) in rodents has been used extensively to decipher the molecular and cellular clues of liver regeneration. During this process, the liver regenerates through hepatocytes, without the help of a stem cell compartment. A particularly fascinating point about this process is that near all quiescent and differentiated hepatocytes quit the G0 phase in a tightly synchronous manner to progress into the G1/S phase transition and replicate their DNA. This massive coordinated entry into the cell cycle is illustrated by a sharp peak of BrdU (Bromodeoxyuridine) incorporation whose timing differs among species (24 hours in rats and 36 to 42 hours in mice), reflecting the variability in the length of the G1 phase. Even if hepatocyte S phase entry is tightly synchronized, hepatocyte replication starts from periportal area and progresses rapidly towards perivenous area. Other nonparenchymal cells such as stellate cells, biliary and endothelial cells proliferate after hepatocytes, responding potentially to other signals.

This paper will focus on the molecular mechanisms involved in this synchronous entry into the cell cycle, highlighting the specific role of the epidermal growth factor receptor (EGFR) during this process in all its complexity.

## 2. Growth Factors and the Synchronous Entry of the Hepatocytes into the Cell Cycle

Hepatocyte proliferation is preceded by an inflammatory stimulus, described in the pioneering work of Nelson Fausto as the “priming phase” [1, 2]. This first step is reversible since, without the subsequent involvement of growth factors, hepatocytes do not progress through cell cycle and return to quiescence. It involves the secretion of cytokines by nonparenchymal cells such as Kupffer cells and poises hepatocytes to become receptive to these growth factors [3, 4]. *In vivo*, this priming stage is required since hepatocytes exhibit only a minimal response to transforming growth factor alpha (TGF-*α*), epidermal growth factor (EGF), or hepatocyte growth factor (HGF) without it. In contrast, these factors are potent mitogens *in vitro *[5–8]. In primary culture, hepatocytes replicate their DNA synchronously after addition of EGFR ligands, suggesting that isolation of hepatocytes from the liver induces priming [6, 9, 10] and for review [11–13].

After cytokines have triggered the G0 to G1 phase transition, required growth factors for the progression through the cell cycle into the S phase are signaling through two main tyrosine-kinase receptors: EGFR and c-Met. 

HGF is the main ligand of c-Met receptor. It is mainly secreted by macrophages and endothelial liver cells [14]. Overexpression of HGF in the liver of transgenic mice increases hepatocyte proliferation during postnatal development and accelerated liver regeneration after PH but has minor effects at adult stage in a quiescent liver [15–17]. On the contrary, conditional deletion of this receptor, as well as studies using RNAi in the liver of mice, caused either a significant decrease in the peak of proliferation [18, 19] or a delay of S-phase entry [20]. Moreover, Thorgeirsson's team indicated that c-Met is required for G2/M progression as well as entering the cell cycle *in vivo *[19].

As opposed to HGF/c-Met axis, EGFR has numerous ligands (EGF, amphiregulin, HB-EGF, TGF-*α*, epiregulin, betacellulin, epigen). For this reason, the implication of this pathway after PH has been studied extensively throughout the years, as it involves several growth factors and downstream pathways to control the proliferation balance. Interestingly, Mitchell et al. showed that after 1/3 PH in mice, there is a lack of a synchronous wave of DNA replication [21]. They then observed that, among the growth factors induced during liver regeneration, the secretion peak of HB-EGF usually observed 24 h after 2/3 PH was absent after the 1/3 PH [21]. HB-EGF injection in 1/3 hepatectomized mice is then sufficient to restore a peak of BrdU incorporation in hepatocytes [21]. Besides highlighting the robust mitogen potential of HB-EGF, this study indicated the importance of the EGFR pathway in the synchronous induction of DNA replication in a dose-dependent manner after PH.

## 3. EGFR Pathway

### 3.1. General Description of EGFR

The Epidermal Growth Factor Receptor (EGFR), also known as ErbB-1, is a plasma membrane glycoprotein, which belongs to the ErbB family of receptor tyrosine kinases (RTKs) jointly with ErbB-2, ErbB-3, and ErbB-4 [22]. It contains an extracellular domain with two cysteine-rich regions, a single transmembrane-spanning region, and a well-conserved cytoplasmic tyrosine kinase domain [23]. Upon ligand binding, ErbB proteins can either homo- or heterodimerize with other members of the ErbB family to activate downstream signaling pathways that regulate proliferation, growth, and differentiation [24]. 

**Figure 1:**
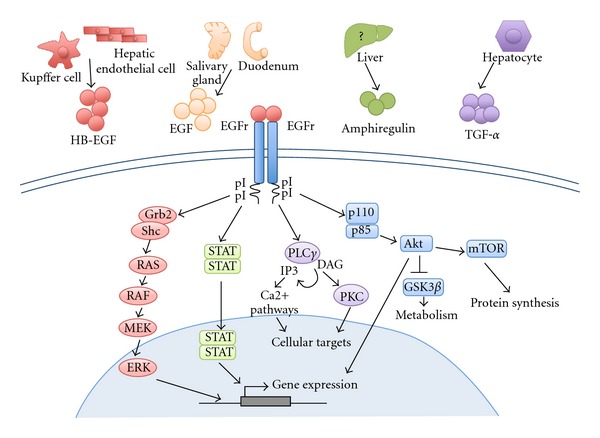
EGFR induced signaling pathways. The major source of each EGFR ligands involved in liver regeneration is schematized. Amphiregulin liver induction right after PH is not sufficient to determine the cellular origin of this secretion. Upon binding of its ligands, EGFR homodimerizes leading to phosphorylation of many tyrosine residues localized in the carboxy-terminal tail of EGFR. Phospho-EGFR is then able to recruit adaptor proteins. They transduce the EGFR signaling by inducing several EGFR-dependent pathways, including the RAS-MAPkinase, PI3K-AKT, PLC*γ*, and Stat pathways. Collectively these pathways control proliferation, differentiation, migration, and survival of the cell.

EGFR was the first member of this family as well as the first RTK to be discovered [25] and plays an essential role in the development of epithelial cells but also in tumors of epithelial cell origin [26]. Ligand induced EGFR dimerization leads to receptor autophosphorylation at tyrosine residues (Figure 1). Some of them can be regulated via other signals like growth hormone [27, 28] or oxidative stress [29, 30]. Phosphotyrosine residues allow the recruitment of specific partners to activate different downstream pathways. EGFR controls a variety of signals ranging from cell proliferation, cell motility, apoptosis decrease, to epithelial-mesenchymal transition, upregulation of matrix metalloproteinases, and has even been proposed to be involved in stem-cell maintenance [31]. Moreover, EGFR has also been shown to regulate downstream targets by directly translocating its internal region into the nucleus, activating cell cycle genes such as Cyclin D1 [32] or genes involved in inflammation like COX-2 [33]. Interestingly, Cox-2-deficient mice showed an impaired liver regeneration [34].

EGFR signaling is regulated in part by endocytic sorting [35, 36]. Upon ligand binding, EGFR is internalized and trafficked to the endosome. Depending on ligand/EGFR complex stability [37] and ubiquitination process by cbl family proteins [38], EGFR is either degraded in the lysosomal compartment or recycled to the plasma membrane [35, 37, 39]. This process may represent an important negative feedback regulatory mechanism to control EGFR signaling [35, 36].

### 3.2. EGFR Pathway in the Liver

There is a strong expression of EGFR in the adult liver, but also during development and regeneration, suggesting an important role for its function [40]. Disruption of EGFR in mice has led to death from mid-gestation up to third week depending on the genetic background, showing various signs of abnormalities to multiple organs including the skin, kidney, brain, gastrointestinal tract, and the liver with thickened hepatocyte cords, distorted sinusoidal anatomy, and abnormally vacuolized nuclei [41]. Specific deletion of EGFR in hepatocytes did not reveal any phenotypical abnormality apart from a reduction in body weight [42]. It has been shown that EGFR ligands exhibit functional differences in models of paracrine and autocrine signaling [43]. Several ligands, such as amphiregulin, epidermal growth factor (EGF), heparin-binding EGF (HB-EGF), betacellulin, epiregulin, and TGF-*α* have been shown to be able to activate the EGFR pathway and some of them induced strong mitogens signals in the liver [44]. There is no evidence that these ligands bind specifically to EGFR and not to other ErbB proteins with whom EGFR can dimerize, although their essential role during liver regeneration has been demonstrated for some of them as described below [45].

There are four main downstream pathways usually associated with EGFR activation: Ras/MAPK, PI3K/Akt, signal transducer and activator of transcription (Stats) and phospholipase C-gamma 1 (PLC*ϒ*1) pathways [46] (Figure 1). However, it has been shown in different epithelial cell types *in vitro* that ligands binding to EGFR induce different downstream signaling pathways according to their affinity. While high affinity ligands (10% of EGFR pool) activate Ras/MAPK and PI3K/Akt pathways, low affinity ligands (90% of EGFR pool) induce Stats and PLC*ϒ*1 pathways [47]. It is now clear that those different pathways are highly interlinked, but for the following they will be described separately. 

Once activated, the internal region of EGFR can serve as a docking site for Src homology 2 domains such as Grb2 and Shc [48, 49]. Grb2 or Shc then interacts with Ras, leading to an interaction with Raf, which will in terms activate the whole MAP kinases pathway [48–50]. The activation of EGFR can also provide a docking site for p85, which is the protein subunit of PI3K. Once activated, it will in turn phosphorylate Akt to promote cell survival and proliferation [51]. In the liver, EGFR-dependent Stats activation does not depend upon JAK kinases activation, as it is usually the case. Instead, Stats have been proposed to be constitutively associated to EGFR, becoming active directly by EGFR phosphorylation [49, 52]. More recently, the Src-kinase has been proposed to activate Stats through EGFR activation [53, 54]. The precise mechanism of PLC*ϒ*1 activation remains unclear, but it appears that PLC*ϒ*1 is also directly associated to EGFR but does not need tyrosine phosphorylation [55]. The activation of PLC*ϒ*1 will yield 1,2-diacylglycerol (DAG) and inositol 1,4,5-triphosphate (IP3). DAG can then activate PKC whereas IP3 can activate Ca^2+^-dependent pathways [56].

## 4. EGFR Pathway during the G1-S Phase in Hepatocytes

### 4.1. The Input of EGFR Ligands in Invalidation Mouse Models

The mitogenic action of the EGFR signal was first determined *in vitro*, on primary culture of hepatocytes. EGFR ligands were added in serum-free medium culture and tested for their capacity to induce hepatocyte proliferation in rodents. Four of them: TGF-*α*, HB-EGF, EGF, amphiregulin, were determined as hepatocyte growth factors since they allow their synchronized S phase entry [5–8].

After 2/3 PH, the protein level of these EGFR ligands increases rapidly [8, 57, 58]. Their mitogenic role was studied *in vivo* using ligand injection, gene overexpression, RNA interference, and conditional gene knockout strategies.

Loss of HB-EGF expression by knocking-out the gene led to major impairment of liver regeneration characterized by the absence of hepatocytes synchronized S phase entry [21]. Conversely, liver HB-EGF overexpression in transgenic mice induced a drastic increase of proliferating hepatocytes compared to wildtype nontransgenic littermates [59]. 

Salivary glands ablation in rodent [60–62], which are the main source of EGF, provoke a main liver regenerative defect hepatocytes being blocked in G1 phase, as it is the case in the conditional amphiregulin knockout mice [8]. After salivary glands ablation, EGF injections restore hepatocyte proliferation in rats [60–62].

Thus, misregulation of these three latter EGFR ligands leads to the same profile of liver regenerative defect characterized by a desynchronized S phase entry of hepatocytes. These results then suggest that these ligands are not redundant during liver regeneration. In contrast to these three liver EGFR ligands, gene inactivation of TGF-*α* in mice had no effect on liver regeneration [63] although it has been demonstrated on hepatocytes primary culture, that TGF-*α* has the same mitogen capacities as EGF, amphiregulin or HB-EGF [5, 6, 8, 64–66].

Different non-exclusive hypotheses can be proposed to understand the non-redundancy of HB-EGF, EGF and amphiregulin ligands. This can be explained by the importance of ligands sequential binding, by different ligands activating different EGFR downstream pathways or by the necessity to reach a threshold of total ligands quantity, in order to induce proliferative signals. Regarding TGF-*α*, the paradoxical results obtained *in vitro* and *in vivo,* can be explained if we hypothesize that already expressed EGFR ligands compensate for TGF-*α*. Genetic replacement of one ligand by another one at the same physiological level using knocking-in strategies, could help to understand these discrepancies.

### 4.2. The EGF Receptor during Regeneration

The role of EGFR (ErbB1) in the G1 phase of the cell cycle in hepatocytes has also been studied *in vivo* either by RNA interference injection in rats [67] or by conditional gene inactivation in mice [42]. Both experiments induced a major impairment of liver regeneration resulting in an altered progression into the G1 phase. However, mutant livers can finally complete regeneration suggesting that EGFR is a critical regulator of hepatocyte proliferation in the initial phases of this process. As opposed to other tissues, ErbB-2 and ErbB-4 are not expressed in the regenerating liver and thus, cannot heterodimerize with EGFR [40]. However, in contrast with liver regeneration, it has been observed that ErbB-2 is re-expressed in primary culture of hepatocytes, participating to the induction of proliferation *in vitro *[68, 69]. ErbB-3 is induced after PH but its ligands are not known to participate to hepatocyte proliferation [40].

The common molecular mechanism between all these studies consists in a downregulation of cyclin D1 expression, the first cyclin that is activated during progression in G1 phase [70, 71]. However, there is very little information on signaling pathways activated downstream of EGFR in hepatocytes to induce their proliferation. In primary culture of hepatocytes, Erk1/Erk2 and PI3K/AKT cascades have been shown to be activated by EGF to induce hepatocyte proliferation [72, 73]. However, during liver regeneration of EGFR knock outs specifically in hepatocytes in mice, none of these canonical downstream pathways were found dysregulated [42], while livers of rats injected with an EGFR RNAi showed a Stat3 misregulation [67]. Regarding knock outs experiments, the authors only reported a defect in the NF-*κ*B and in p38 activation during the G1 phase [42]. The essential role of NF-*κ*B during liver regeneration is to “prime” hepatocytes and not to participate to the G1 phase progression [4, 10]. However it was suggested that it could control cyclin D1 transcription [74, 75]. *In vitro* studies showed that EGFR could activate Ca^2+^ dependent pathways such as RaI and NF-*κ*B through the phosphorylation of PLC*γ*. PLC*γ* is one of the possible downstream pathways activated by EGFR. It has been shown that the increased activity of nuclear PLC in regenerating rat occurs before DNA synthesis peak after PH [76]. Moreover, Farrell's group pointed out the role of the EGFR/PLC*γ* axis in hepatocyte proliferation in a model of chronic ethanol consumption [77].

In rats injected with RNAi directed against EGFR, Stat3 transcription downregulation was observed by transcriptomic approach [67]. This result may be relevant since Stat3 is a target of EGFR and has the capacity to activate proliferation through cyclin D1 in other cell types. However, it would have been interesting to check both the protein expression and activation level of Stat during the regenerating process, as this hypothesis does not match with cell culture experiments. It was indeed demonstrated that the Stats (Stat3 and Stat5) were not recruited for EGFR dependent hepatocyte proliferation *in vitro* [78].

The discrepancy between the *in vitro* and *in vivo* results makes it difficult to fully understand which are the intracellular targets required to induce the EGFR dependent progression into the S phase.

## 5. Regulation of the EGFR Pathway during Liver Regeneration

### 5.1. Regulation of the Ligands

Liver regeneration efficiency could indeed be controlled either by EGFR ligands induction and/or by EGFR activation. While various factors have been shown to regulate EGFR ligands in the quiescent liver, very little is known during the regenerative process when these factors are induced.

The Hippo signaling pathway, well known to be involved in cell proliferation, could also regulate the EGFR pathway through its pivotal effector, YAP (Yes-associated protein). Indeed, it has been shown that YAP regulates amphiregulin at a transcriptional level [79]. Interestingly, one study showed that YAP protein level increases after PH, suggesting a role in liver regeneration [80]. Loss of Hippo signaling in the mouse liver has been shown to lead to YAP induction and liver hyperplasia with hepatocytes progenitors proliferation [81–83]. However, no study has been yet performed after partial hepatectomy in YAP knockout mice to comfort this potential role.

Different members of the ADAM family induce the maturation of EGFR ligands, by cleaving them, and thus increasing their biodisponibility for EGFR binding [84]. ADAM 10 is able to cleave EGF transmembrane precursors [85]. ADAM 17, also known as tumour necrosis factor-*α*-(TNF-*α*-)converting, enzyme, or TACE, can shed amphiregulin, TGF-*α* and HB-EGF precursors [86, 87]. It has been suggested that ADAM17, upon TNF-*α* addition in hepatocyte cell culture, transactivates EGFR by cleaving TGF-*α*, increasing hepatocyte proliferation [88]. As for liver regeneration, ADAM 17 expression increases at the late G1-phase corroborating a potential regulator role of EGFR signaling during this regenerative process [89].

### 5.2. Regulation of the Receptor

Regarding the receptor, we recently pointed out the major role played by the growth hormone (GH) pathway to control EGFR. GH is a pleiotropic hormone that plays a major role in proliferation, differentiation, and metabolism via its specific receptor. It has been previously suggested that GH signaling pathways are required for normal liver regeneration [90, 91]. Consequently, we recently investigated the mechanism by which GH controls liver regeneration. GH receptor knockout mice (GHrKO) showed a major liver regeneration impairment correlated with a downregulation of ERK1/ERK2 activation [92]. We showed that GH controlled the EGFR expression at the mRNA level in liver from quiescent stage until the mid G1-phase [92]. Most of GH physiological effects are mediated by the Stat5 transcription factor. Interestingly, EGFR expression was drastically down regulated in the liver of mice deleted for Stat5b in their hepatocytes [93]. However, chromatin immunoprecipitation experiments failed to demonstrate that Stat5b binds to EGFR promoter and suggested that it acts indirectly through intermediate proteins [93]. IGF-1, the major target gene of GH/Stat5b axis in the liver could have been an interesting target since it is known to be involved in liver regeneration control and it is drastically downregulated in GHrKO mice [91, 94]. However, we demonstrated that it is not the case, its forced expression in GHrKO mice hepatocytes failing to rescue EGFR expression (personal data). Interestingly, GH has also been described to control EGFR at posttranscriptional level, inducing its phosphorylation in quiescent liver [27, 28]. Accordingly with these data, EGFR failed to be activated by phosphorylation throughout the G1 phase in the hepatectomized GHrKO mice, even when it was reexpressed in mid/late G1-phase [92].

Bile acids that have been shown to contribute to liver regeneration have recently been considered as an intermediate in the interplay between EGFR and the Fas apoptotic pathway. Indeed, CD95L and hydrophobic bile acids are known to transactivate EGFR, but depending on the cell type, CD95-EGFR-mediated signalling ends up in cell apoptosis or cell proliferation. Thus, EGFR activation by CD95L or bile acids can lead to hepatic stellate cell proliferation but hepatocyte apoptosis [95].

Finally, the Wnt/*β*-Catenin pathway that is activated during the mid G1-phase during liver regeneration process could also participate to EGFR regulation during the liver regeneration process [96, 97]. *β*-catenin has been proposed to control EGFR in quiescent liver at a transcriptional level [98], but there is no clear evidence for a direct action of *β*-catenin via the putative Lef/Tcf site present on the EGFR promoter [98]. However, liver regeneration studies on mice deleted for ctnnb1 (the gene coding for *β*-catenin), although leading to a liver regeneration delay, did not point out an EGFR expression impairment [96, 97, 99]. We can hypothesize that *β*-catenin pathway compensates for EGFR defect in GHrKO mice from mid G1-phase when EGFR expression was reinduced.

## 6. Conclusion

Altogether, these data highlight the major role played by growth factors via EGFR in the liver regeneration process. Its activation during the G1 phase controls the cell cycle progression of hepatocytes from the G1 phase until the S phase leading to the synchronized hepatocytes S-phase entry. In liver regeneration, even though ligands have been identified, downstream pathways leading to hepatocytes S phase entry as well as the mechanisms that regulate EGFR pathway activation remain to be determined. In this context, our results point out the major role played by GH to control its expression and activation during the regenerative process [92].

 The degree of complexity of hepatocyte proliferation's regulation by growth factors is reinforced by the question of a potential crosstalk and/or redundancy between EGFR and HGF pathways since they both induce hepatocytes G1/S progression and can activate the same downstream gene cascades [18–20, 42, 67]. A relationship between these pathways has been suggested by the observation that loss of c-Met or EGFR both lead to major liver regeneration impairment. This could result from the necessity of two independent pathways or by the existence of an essential interrelation between both pathways, to induce a robust hepatocyte proliferation signal. Since *in vitro* studies led to discordant results, it should be interesting in the future to test the redundancy or independence of c-met and EGFR pathways for liver regeneration in double knockout mice.

Given the importance of EGFR signaling to control hepatocytes division and its regulation by GH, it will be interesting to determine the incidence of misregulations of the GH/EGFR axis on the liver proliferative capacity in hepatic physiopathology. There have been numerous studies in human and in mice reporting defects of the GH signaling in various liver pathologies. For example, liver cirrhosis has been associated with the inhibition of GH signaling in the liver [100, 101]. Obesity, often associated with hepatic steatosis and insulinoresistance, is also characterized by a decrease of GH level in the serum of patients [102, 103]. We found in different mouse models that hepatic steatosis is associated both with a downregulation of GH pathway and a downregulation of EGFR expression (personal data). We therefore hypothesize that the loss of liver proliferation capacity in liver steatosis is related to the GH/EGFR axis misregulation.

In contrast, the EGFR signaling upregulation has been involved in cancer development in many tissues [104]. In hepatocellular carcinoma, its misregulation was found in 60 to 80 percent of patients, depending on studies, leading to the suggestion that EGFR signaling upregulation was associated with the increased proliferative capacity of liver tumoral cells [105].

The complete deciphering of EGFR signaling regulatory pathways resulting in this tricky balance will therefore be crucial in the future to develop appropriate therapeutic strategies allowing stimulation of hepatocyte proliferation in chronic liver diseases if required or in contrast, to reduce it in cases of tumoral progression.
